# Absolute Consistency: Individual versus Population Variation in Annual-Cycle Schedules of a Long-Distance Migrant Bird

**DOI:** 10.1371/journal.pone.0054535

**Published:** 2013-01-16

**Authors:** Jesse R. Conklin, Phil F. Battley, Murray A. Potter

**Affiliations:** Ecology Group, Institute of Natural Resources, Massey University, Palmerston North, New Zealand; University of Western Ontario, Canada

## Abstract

Flexibility in scheduling varies throughout an organism’s annual cycle, reflecting relative temporal constraints and fitness consequences among life-history stages. Time-selection can act at different scales, either by limiting the range of alternative strategies in the population, or by increasing the precision of individual performance. We tracked individual bar-tailed godwits *Limosa lapponica baueri* for two full years (including direct observation during non-breeding seasons in New Zealand and geolocator tracking of round-trip migrations to Alaska) to present a full annual-cycle view of molt, breeding, and migration schedules. At both population and individual scales, temporal variation was greater in post-breeding than pre-breeding stages, and greater in molts than in movements, but schedules did not tighten across successive stages of migration toward the breeding grounds. In general, individual godwits were quite consistent in timing of events throughout the year, and repeatability of pre-breeding movements was particularly high (*r* = 0.82–0.92). However, we demonstrate that *r* values misrepresent *absolute* consistency by confounding inter- and intra-individual variation; the biological significance of *r* values can only be understood when these are considered separately. By doing so, we show that some stages have considerable tolerance for alternative strategies within the population, whereas scheduling of northbound migratory movements was similar for all individuals. How time-selection simultaneously shapes both individual and population variation is central to understanding and predicting adaptive phenological responses to environmental change.

## Introduction

Migration is an adaptive response to seasonally changing resources, and thus selection for appropriate timing is considered a key driver of life-history evolution in migratory birds [1], [2]. For some annual life-history stages [3], timing has a clear fitness consequence; a well-documented example is the importance of arrival timing on breeding grounds for subsequent reproductive success [4], [5]. Timing of other stages (e.g., post-breeding molt) may have less obvious fitness consequences, but can yet be subject to time-selection if carry-over effects lead to cross-seasonal interactions between performance in stages across the annual cycle [6], [7]. In migratory birds, we generally expect pre-breeding stages to be more time-selected than post-breeding stages [8], and temporal constraints to increase with proximity to the breeding season [9], but empirical evaluations of these hypotheses are hampered by the paucity of detailed year-round data for migratory populations.

Time constraints may exist at the population or individual level, or both. At the population level, increasing time-selection should reduce the range of alternative strategies tolerated by the system [10], [11], leading to decreased inter-individual variation. At the individual level, time-selection should increase the precision with which individuals enact these strategies; this predicts decreased intra-individual variation in timing if relevant conditions are stable, but in variable conditions the ‘optimal’ response may be flexibility [12]. Ultimately, performance in any particular stage depends not just on the costs of sub-optimal performance (i.e., selection pressure), but also an individual’s ability to behave optimally, which may vary with control mechanisms [3], [13], environmental conditions [14], and individual quality [15].

Repeatability (the intra-class coefficient, *r* [16]) is an increasingly popular metric in migration studies concerned with the consistency and flexibility of individual life-history scheduling [17]. However, because *r* is a product of both inter- and intra-individual variation, it is best used as a comparative index when both of these parameters are well-described by samples in multiple annual stages. Due to the logistical constraints of following individual migratory birds across multiple years, studies to date have calculated *r* values for a large sample of individuals in one or few consecutive stages (e.g., [18], [19]), which limits inference across seasons, or for movements of a small sample of remotely-tracked individuals (e.g., [20]), which is unlikely to fully describe population variation. Understanding the significance of variation in scheduling among life-history stages (and the utility of *r* values to characterize this) requires multi-year individual data across the entire annual cycle.

**Figure 1:**
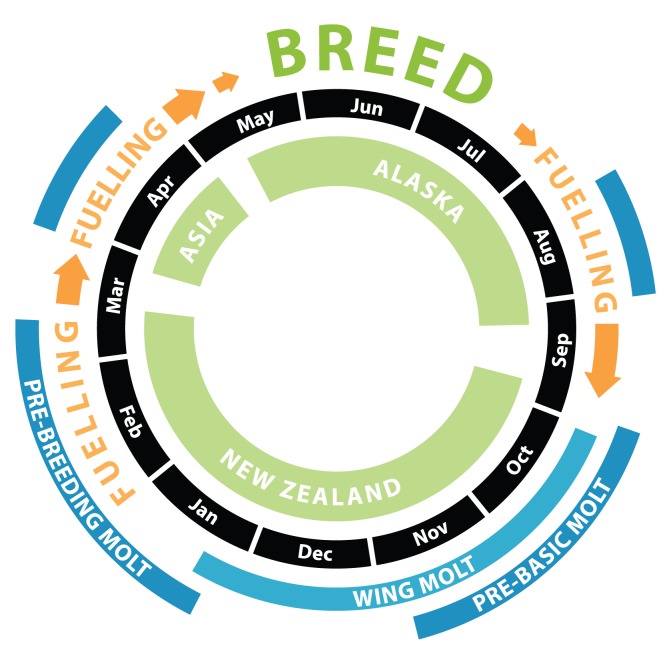
Generalized cycle of annual life-history stages of New Zealand bar-tailed godwits. Typical adult male routine is shown. Large arrows indicate major migratory flights. Smaller arrows indicate movements within Alaska. Pre-basic and pre-breeding contour molts are suspended during migratory flights.

The annual routine of New Zealand bar-tailed godwits *Limosa lapponica baueri* (Figure 1) includes a short, high-latitude breeding season [21], a complex molt [22], [23], and the two longest non-stop migratory flights yet recorded [24], [25], and thus may feature crucial time constraints and trade-offs among life-history stages. In this study, we combine two years of detailed observations of color-banded bar-tailed godwits in New Zealand with geolocator-tracking of a subset of the same individuals, to describe timing of life-history stages throughout the annual cycle, including molts, movements, and breeding. At both the population and individual levels, we test four predictions: (1) temporal variation decreases throughout the year from post-breeding movements to the following breeding season; (2) temporal variation decreases through successive stages of spring migration; (3) timing of pre-breeding movements is more rigidly maintained than that of post-breeding movements; and (4) temporal variation in molts is greater than in migratory movements. We discuss the implications of population versus individual variation among life-history stages for relative time-selection and tolerance for alternative strategies, and the utility of repeatability values to assess the consistency of individual behavior.

## Methods

### Ethics Statement

Fieldwork was conducted with Massey University Animal Ethics Committee approval (#07_163).

### Individually Marked Birds

During three non-breeding seasons (January–April 2008, September 2008–April 2009, and September 2009–April 2010), we studied molt and migration timing in a small population of bar-tailed godwits (200–280 godwits; approximately 25% were individually color-banded) at the Manawatu River estuary, New Zealand (40.47°S, 175.22°E). We conducted high-tide surveys every 3–4 days (d) during migratory arrival (1 September–20 October), daily during migratory departure (4 March–5 April), and every 4–8 d during the intervening summer months (21 October–3 March). During surveys, we used direct observation and digital photography to monitor primary feather molt, contour feather molt, and presence/absence of all marked individuals (77 total; 35 male, 42 female; *n* = 58–63 per season). With these data, we determined for each individual the timing of migratory arrival, completion of pre-basic contour feather molt, initiation and completion of primary feather molt, initiation of pre-breeding contour feather molt, and migratory departure. Departures were generally observed directly, and other parameters were estimated to within approximately 1–6 d. We have described details of data collection and analysis specific to each parameter elsewhere: arrival and contour molt [22], primary molt [23], and departure [26].

### Light-level Geolocators

A subset of color-banded godwits at the site was additionally equipped with leg-mounted light-level geolocators (British Antarctic Survey model MK14; 1.4 g; 2-year life) to track movements outside of New Zealand. Twenty instrumented individuals (9 male, 11 female) provided data for this study; eight of these were tracked for two entire annual cycles. Derived breeding locations of these godwits spanned most of the known breeding range in Alaska (59–70°N [21]), indicating that our sample encompassed most variation in migration schedules present in the greater New Zealand population.

The geolocators recorded sunrise and sunset, allowing daily calculation of latitude and longitude (±130 km error, based on ground-truthing units and resightings of instrumented godwits [27]), except during ±15 d of the vernal or autumnal equinox, when only longitude is reliable [28]. To derive fuelling and breeding sites outside New Zealand, we averaged twice-daily locations over periods when birds were relatively stationary, excluding clear outliers likely resulting from weather- or behavior-related shading events near dawn or dusk. Clear shifts in latitude or longitude indicated the initiation of major migratory movements, which we considered concluded when the bird’s location once again stabilized. Sample sizes decreased throughout May–September due to unit failures. Additionally, we could not determine timing of departure from the breeding area for two birds, because their breeding and post-breeding staging locations differed by less than the location error of the geolocators.

Geolocators also indicated periods of nest incubation [21], [29]. During the breeding season, geolocators registered nights as regular, clearly demarcated periods of darkness <4.5 hours in length; these did not appear at all if birds bred north of 64°N. Days appeared as continuous light, irregularly broken by brief (<1 hour) shading events, most likely corresponding to behaviors such as wading or sitting. Within 6–25 d of apparent arrival on breeding grounds, most birds (14 of 16 cases) displayed a conspicuous pattern of incubation, in which semi-regular shading events of 4–13 hours were overlaid on the day/night pattern for periods up to 25 d. We considered the first day of this period to be the start of incubation.

### Analysis

We present data for two complete annual cycles, from New Zealand departure in 2008 through the initiation of pre-breeding contour molt in 2010 (the period for which geolocator data are available); this includes geolocator data for events outside New Zealand, and direct observations of color-banded godwits (including the geolocator-tagged birds) for events within New Zealand. To describe population variation in timing of each stage, we pooled all observed dates across two annual cycles (1–2 observations per bird) and calculated total population span (the difference between the earliest and latest individuals) and standard deviation (SD). To describe intra-individual variation in timing for each stage, we used the difference (d) between the two values for each individual observed in both years, and calculated the mean and SD across all individuals. We tested for differences among stages using Kruskall-Wallis non-parametric ANOVA and Dunn’s post-hoc pairwise comparison tests.

For each stage, we calculated repeatability (intra-class correlation coefficient, *r* [30] ±SE [31]) of individual timing for all godwits with two years of data. For comparison, we calculated *r* separately for all color-banded godwits and the subset of geolocator-tagged birds. We considered *r* values to differ significantly if the 95% confidence intervals [16] did not overlap.

To ask whether temporal variation decreased across all annual life-history stages (*n* = 13), and across stages of spring migration (*n* = 5), we ranked data across stages (including partial ranks for ties) and tested for differences from hypothesized ranks (stages ranked chronologically from post-breeding dispersal to first incubation) using Spearman-rank correlation (one-tailed). We used one-tailed Wilcoxon signed-rank tests to compare variation between post-breeding (*n* = 3) and pre-breeding movements (*n* = 5), and between movements (*n* = 8) and molt parameters (*n* = 4).

In this population, individual schedules are linked with breeding site phenology: due to relative timing of spring thaws, godwits breeding in northern Alaska migrate later than southern breeders in both spring and autumn [21], and timing of molts show corresponding differences [22], [23]. To estimate the expected inter-individual variation in all annual stages based on breeding latitude, we examined snow-melt phenology data (2000–2012 [32]) across the godwits’ Alaska breeding range [33], and calculated for each year the difference (d) between the earliest snow-free days in the southern end of the breeding range (Yukon-Kuskokwim Delta; 59.7–63.3°N) and the northern end of the range (North Slope of the Brooks Range; 69.6–71.3°N).

### Results

**Figure 2:**
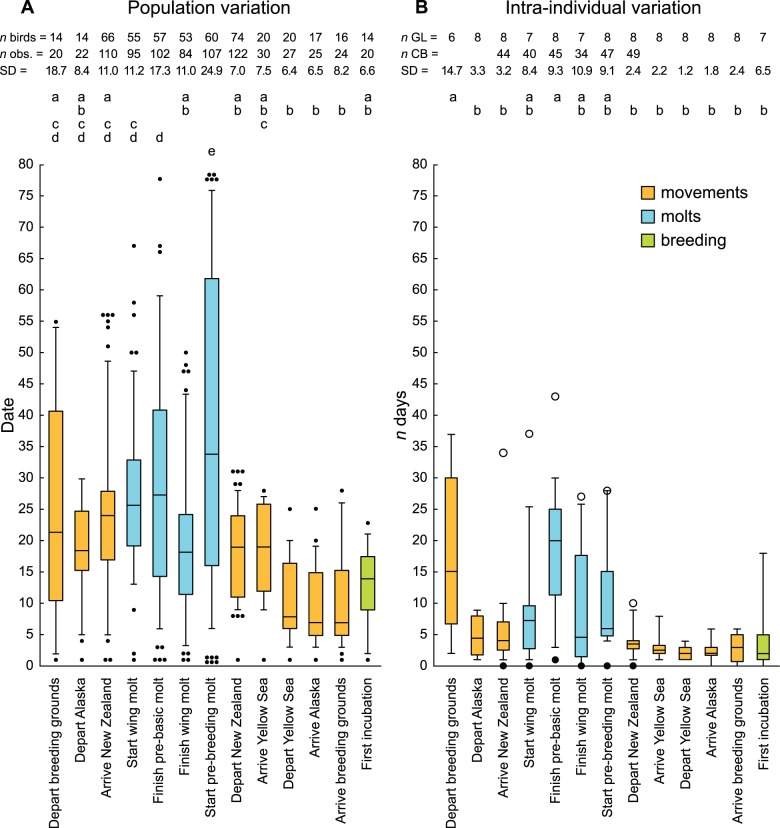
Population and individual variation in timing throughout the annual cycle of bar-tailed godwits. Stages (*x*-axis) are in chronological order from left to right, starting with dispersal from breeding grounds (see Figure 1). (A) Distribution of all observations during March 2008*–*March 2010, standardized by date (day 1 =  earliest observation for each stage). Data are derived from color-banded (New Zealand only) or geolocator-tracked (outside New Zealand) godwits. Boxplots indicate median and 25^th^ and 75^th^ percentiles; whiskers indicate 5^th^ and 95^th^ percentiles; filled circles indicate more extreme values; overlapping points are offset for clarity. Standard deviation (SD) is shown above. (B) Intra-individual variation in timing (difference between dates in successive years) for geolocator-tracked godwits (GL; sample sizes = individuals observed in two years). Boxplots indicate median and 25^th^ and 75^th^ percentiles; whiskers indicate entire range of values; SD is shown above. Circles indicate greatest (open) and least (filled) within-individual differences in the larger sample of color-banded (CB) birds (New Zealand only; sample sizes shown). Letters indicate significant differences in Kruskal-Wallis test followed by Dunn’s post-hoc comparison.

To see how well our geolocator sample represented variation in the larger population, we tested for statistical differences from color-banded samples when both types of data were available (six stages within New Zealand). The larger color-banded samples naturally contained more extreme values (Figure 2B), but medians and distributions of values in every stage were similar to geolocator samples for both population (Mann-Whitney tests, all *P* = 0.25–0.88) and individual data (all tests *P* = 0.18–0.96). Therefore, we combined the two data sources for the best representation of population variation throughout the year. However, we limited analyses of individual variation to geolocator birds tracked for two entire annual cycles (*n* = 8), to ensure that samples for all stages were as comparable as possible.

Population variation differed among annual stages (Kruskal-Wallis; *H* = 165.9, df = 12, *P*<0.0001); depending on stage, the total population span (difference between the earliest and latest individuals) varied from 23 to 78 d (Figure 2A). Intra-individual variation also varied among stages (*H* = 33.2, df = 12, *P* = 0.0009); however, despite median annual differences ranging 2–20 d, small samples provided low power to distinguish among specific stages in post-hoc tests (Figure 2B).

**Figure 3:**
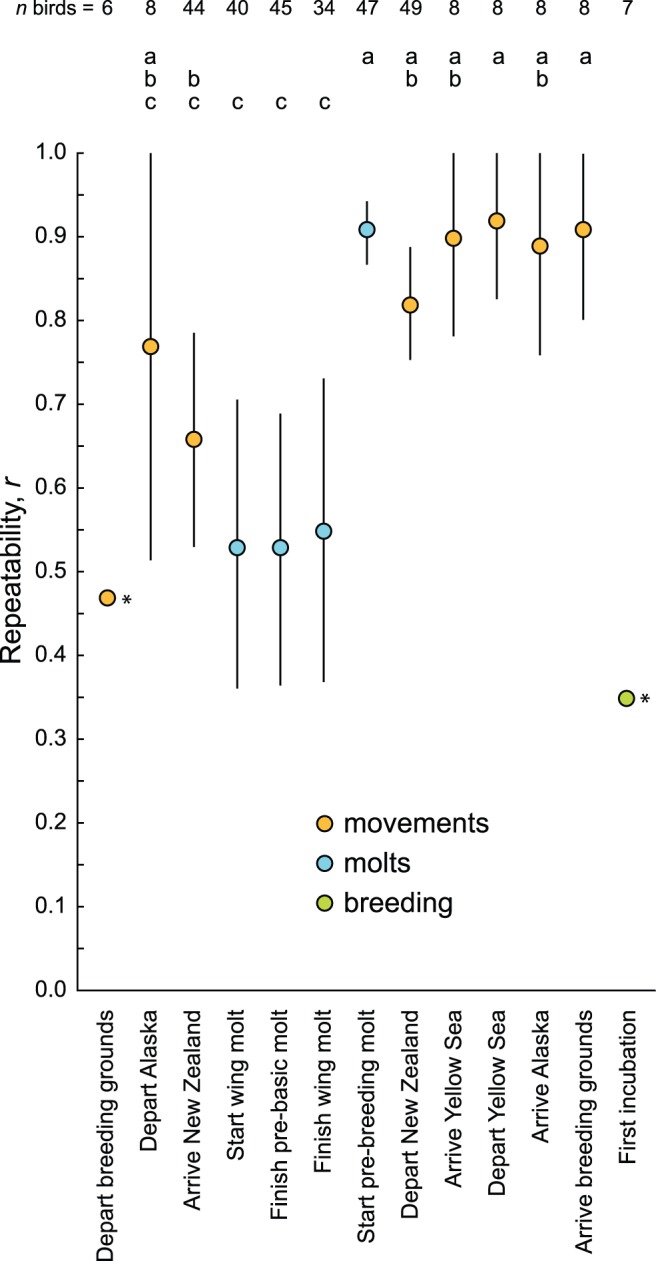
Individual repeatability in timing throughout the annual cycle of bar-tailed godwits. Individual repeatability (*r*) among color-banded (New Zealand only) or geolocator-tracked (outside New Zealand) godwits (see Table 1). Whiskers indicate 95% confidence intervals. Asterisks indicate non-significant *r* values. Letters indicate significant differences based on non-overlapping confidence intervals.

Among geolocator-tagged godwits with two years of data, we found significant individual repeatability in 8 of 13 stages (Table 1); repeatability was not significant for 3 of 4 molt parameters, incubation, or departure from breeding sites. The larger sample of color-banded godwits demonstrated significant repeatability in all stages in New Zealand (Table 1). For further comparisons of repeatability, we used the color-banded samples for stages in New Zealand, and the geolocator samples for stages outside New Zealand (Figure 3).

**Table 1:** Individual repeatability in timing of key events in the annual cycle of bar-tailed godwits.

	Geolocators					All color-banded				
Stage	*n*	*r*	SE	*F*	*P*	*n*	*r*	SE	*F*	*P*
Depart breeding	6	0.47	0.26	2.8	0.12					
Depart Alaska	8	**0.77**	**0.11**	**7.8**	**0.005**					
Arrive New Zealand	8	**0.77**	**0.11**	**7.7**	**0.005**	44	**0.66**	**0.06**	**4.9**	**<0.001**
Start wing molt	7	0.44	0.25	2.6	0.12	40	**0.53**	**0.08**	**3.3**	**<0.001**
Finish pre-basic molt	8	0.37	0.25	2.2	0.15	45	**0.53**	**0.08**	**3.3**	**<0.001**
Finish wing molt	7	−0.23	0.52	0.6	0.71	34	**0.55**	**0.09**	**3.5**	**<0.001**
Start pre-breeding molt	8	**0.76**	**0.11**	**7.3**	**0.006**	47	**0.91**	**0.02**	**20.3**	**<0.001**
Depart New Zealand	8	**0.86**	**0.07**	**13.2**	**0.001**	49	**0.82**	**0.03**	**10.3**	**<0.001**
Arrive Yellow Sea	8	**0.90**	**0.05**	**19.3**	**<0.001**					
Depart Yellow Sea	8	**0.92**	**0.04**	**24.7**	**<0.001**					
Arrive Alaska	8	**0.89**	**0.05**	**17.4**	**<0.001**					
Arrive breeding	8	**0.91**	**0.05**	**21.4**	**<0.001**					
First incubation	7	0.35	0.28	2.1	0.18					

Significant results are indicated in bold.

### Temporal Variation throughout the Year

At the population level, temporal variation (SD) decreased chronologically from post-breeding departure to start of incubation (Spearman-rank: *r_s_* = 0.71, *n* = 13, *P* = 0.003). Intra-individual variation also decreased chronologically across all stages (mean: *r_s_* = 0.72, *n* = 13, *P* = 0.003; SD: *r_s_* = 0.59, *n* = 13, *P* = 0.016). Individual repeatability (*r*) increased chronologically from Alaska departure through arrival on breeding grounds (*r_s_* = 0.72, *n* = 11, *P* = 0.006).

### Successive Stages of Spring Migration

We found no evidence that schedules tightened across stages of migration toward the breeding grounds. Temporal variation did not decrease chronologically from New Zealand departure to arrival on breeding grounds at the population level (SD: *r_s_* = 0.20, *n* = 5, *P* = 0.66) or individual level (mean: *r_s_* = 0.36, *n* = 5, *P* = 0.28; SD: *r_s_* = 0.30, *n* = 5, *P* = 0.34). Repeatability did not increase across spring migration (*r_s_* = 0.50, *n* = 5, *P* = 0.23).

### Pre-breeding versus Post-breeding Movements

Mean population spans were 47.0 d (range = 30–55 d) for post-breeding movements and 27.8 d (range = 25–33 d) for pre-breeding movements (Wilcoxon test; *W* = 14.0, *n* = 8, *P* = 0.035). Intra-individual variation was also greater for post-breeding movements than for pre-breeding movements (mean: *W* = 15.0, *n* = 8, *P* = 0.018; SD: *W* = 15.0, *n* = 8, *P* = 0.036); specifically, departure from the breeding grounds was the most variable annual movement by far (Figure 2B). Repeatability was lower for post-breeding movements (mean = 0.72) than for pre-breeding movements (mean = 0.89; *W* = 0.0, *n* = 7, *P* = 0.048).

### Molts versus Movements

Mean population spans were 68.3 d (range = 50–78 d) for molt parameters and 34.8 d (range = 23–55 d) for movements (*W* = 34.0, *n* = 12, *P* = 0.008). Intra-individual variation was also greater for molts than for movements (mean: *W* = 28.5, *n* = 12, *P* = 0.021; SD: *W* = 28.0, *n* = 12, *P* = 0.025). Repeatability was lower for molts (mean = 0.63) than for movements (mean = 0.84), but this difference was marginally non-significant (*W* = 5.0, *n* = 11, *P* = 0.055).

### Components of Population Variation

**Figure 4:**
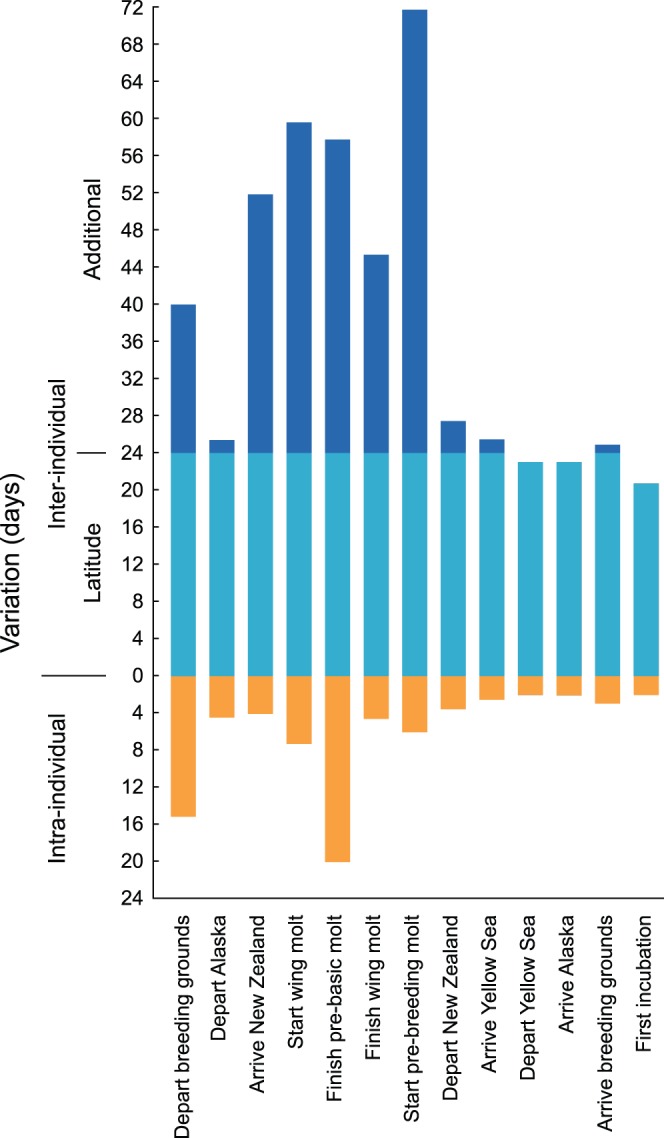
Components of population variation in timing of annual cycle events of bar-tailed godwits. For each stage, bar indicates total span of population variation (see Figure 2A). ‘Intra-individual’ (orange) indicates median individual difference between years (2-year geolocators only; see Figure 2B). ‘Latitude’ (light blue) represents expected inter-individual variation related to breeding latitude (24 d). ‘Additional’ (dark blue) indicates remaining inter-individual variation after subtracting latitude variation.

The total range of variation observed in a particular stage (represented by the population span) comprises all sources of intra- and inter-individual variation (Figure 4). In this study, intra-individual variation represented a relatively small portion of total population variation (median individual difference = 7–27% of population span; mean = 12%).

The greatest single influence on temporal inter-individual variation in this population is known to be breeding latitude. On average, the southern end of the Alaska breeding range became snow-free 24 d (range = 11–38 d, *n* = 13 years) before the northern end. This expected variation based on latitude represented a much greater proportion of population spans (31–104%; mean = 66%; Figure 4) than did intra-individual variation.

After subtracting intra-individual and latitudinal variation, remaining inter-individual variation was dramatically different among stages (0–72 d; Figure 4). In particular, pre-breeding movements featured little or no additional variation (≤3 d), whereas molts demonstrated 20–48 d of additional persistent inter-individual differences.

## Discussion

Although there is a wealth of theoretical support for variation in time-selection across the annual cycle (e.g., [8]), few empirical studies have addressed the timing of migratory movements in more than one season, and none has additionally included scheduling of molts and breeding for a comprehensive view of temporal variation throughout the year. Using a combination of direct observation and geolocator data, we present a unique view of year-round temporal variation for a set of migratory individuals, placed within the context of population-level patterns. These data allow empirical examinations of expected patterns that, despite their fundamental nature, have yet to be properly tested in wild migratory populations. They also allow evaluation of how selection has simultaneously shaped inter- and intra-individual variation across the entire annual cycle.

### Temporal Variation throughout the Year

We have previously reported high consistency of individual timing of departure from New Zealand [18], [26]; this study shows that similar rigidity characterizes much of the godwits’ annual cycle. Median intra-individual differences were <5 d for 9 of 13 annual stages, resulting in several repeatability values that rank among the highest reported for any animal behaviours [34]. By contrast, inter-individual variation was surprisingly high for a presumably time-constrained system; we observed population spans ≥50 d in 6 stages, including all molt parameters.

Although Spearman Rank tests were consistent with decreasing variation throughout the year for population and individual data, temporal patterns clearly deviated from a simple successive incremental tightening with each stage (e.g., molts in New Zealand generally demonstrated more variation than the preceding southbound movements). The hypothesized ideal scenario (A>B>C>D, etc.) implies increasing time-selection with date and to some extent avoidance of temporal carry-over effects among stages, but does not account for differences in relevant control mechanisms and inter-dependency of stages. Timing of some stages is clearly dependent on prior stages (e.g., breeding obviously cannot commence before completion of spring migration), whereas others may be independent of each other (e.g., staging godwits in Alaska suspend pre-basic molt and commence southbound migration, regardless of the proportion of molt they have performed [22]). In this population, migration timing appears to be under fairly strong endogenous control, and relatively unaffected by, for example, duration of prior breeding investment [21] or timing of molts [23].

### Successive Stages of Spring Migration

Contrary to our prediction, schedules did not significantly tighten across stages of northward migration: variation in all stages was relatively low at both population and individual levels, but neither showed a decreasing trend between departure from New Zealand and arrival on the breeding grounds. This finding appears at odds with a number of sandpiper studies showing that individual migration speed increases and stopover duration decreases as the breeding season approaches [9], [10], [35], [36], predicting decreasing population spans across successive stages of migration [37]. However, two aspects of the spring migration of New Zealand bar-tailed godwits may lead to important differences with other species. First, godwits perform two extended (∼5,000–10,000 km) trans-oceanic flights, limiting the range of possible migration strategies, in terms of physiological preparation and the number, location, and duration of stopovers. Second, annual consistency of timing of departure from New Zealand is remarkably high at both the individual and population level [18], [26]; this leaves little room for additional tightening of schedules after initiation of migration.

Another relevant factor general to ‘long-jump’ migrants is that cues for annual variation in breeding phenology are unavailable from the wintering grounds. This means that migration is initiated without regard to environmental variation at the ultimate destination, and this additional source of variation is added later during the journey, leading to movements that are more rigidly timed than breeding itself [38], [39]. Godwits probably receive their first cues regarding breeding phenology upon arrival in southwestern Alaska, and the difference between this stage and their arrival at specific breeding sites likely reflects individuals fine-tuning their schedules according to local environmental cues.

### Pre-breeding versus Post-breeding Movements

Our population and individual data support the view that pre-breeding movements are more time-constrained and repeatable than post-breeding movements. These results are unsurprising, and supported by a wealth of theoretical [8] and empirical studies [40], [41], [42]. More surprising, given the unclear fitness benefits of arrival timing on wintering grounds, is the consistency with which individuals timed their autumn migrations: on average, individuals varied by only 4 d between years on both departure from Alaska and arrival in New Zealand, and repeatability values for this journey (*r* = 0.66–0.77) are greater than most reported values for migration *toward* the breeding grounds [17], [19], [43]. Each year of our study, 92–96% of color-banded godwits arrived in New Zealand within a 32-day span (similar to northbound departure), and the remainder trickled in over the subsequent four weeks; these few stragglers represent both ‘unintended’ detours during the southbound flight (e.g., [24]) and annually consistent individual routines (Conklin and Battley unpubl. data). The autumn migration of Alaskan bar-tailed godwits is unique in that it typically covers 11,000–12,000 km in a single non-stop flight of 8–10 days [24], [25], the timing of which depends primarily on breeding latitude [21] and the occurrence of favorable weather systems [24]. Individual breeding success is likely to drive initial departure from breeding sites, but it does not appear to affect timing of godwits’ departure from Alaska [21]. We expect differences between spring and autumn to be much more profound in systems in which timing or duration of autumn migration is strongly influenced by extent of prior breeding investment (e.g., [44]) or annual variation in conditions at staging or stopover sites [45].

### Molts versus Movements

Greater variation in timing of molts than in movements presumably reflects differences in both regulatory mechanisms and fitness consequences. Migration timing has theoretical and demonstrated consequences for subsequent activities such as breeding [1], [4] and molts [44], [46], and timing of protracted trans-oceanic flights may have direct survival consequences, particularly if wind assistance is necessary for successful migration [47]. Fitness consequences of molt timing are less clear (but see [48]); molt is typically seen as flexible, with birds adjusting both timing and duration to keep to their annual schedules [49], [50]. In this godwit population, within-individual variation in timing of autumn migration carried over to molt schedules in New Zealand, but timing of spring migration was unresponsive to variation in preceding life-history stages [23].

High intra-individual variation in pre-basic molt was as expected; like departure from the breeding grounds (the most flexible annual movement in our study), initiation of this molt is strongly associated with cessation of breeding activity [51], [52], [53], which may vary by more than eight weeks in this population, due to differences in laying date and breeding success [21]. However, the extent of intra-individual variation in pre-breeding molt initiation was greater than expected, given its presumably strict photoperiod control [50] and association with the tightly scheduled spring migration. The cause of this variation is unclear, but it had no apparent effect on subsequent plumage or migration [23].

### Tolerance for Alternative Strategies

For any life-history parameter, the total range of values observed in a population contains two primary components: (1) intra-individual variation resulting from annual differences in performance, and (2) inter-individual variation resulting from persistent differences in strategy or performance. The relative contribution of these sources of variation can only be evaluated in multi-year individual-level studies. For example, a large population window for migratory departures could result from all individuals having a similar strategy but performing inconsistently, or from individuals performing a range of strategies with high precision. There may be only one optimal solution for certain annual tasks, whereas other stages tolerate a number of possible solutions with equivalent fitness consequences. By separating intra- and inter-individual variation in timing across the annual cycle, we can learn how temporal constraints differentially shape behavior at the individual and population levels.

We have attempted to characterize the relative tolerance for alternative strategies among annual stages in Figure 4: after accounting for observed intra-individual variation and known inter-individual differences associated with breeding latitude, the remaining span of observations represents variation resulting from persistent inter-individual differences in performance. These differences may derive from variation in intrinsic ‘quality’ or from negotiation of trade-offs that differ among individuals, perhaps by sex, age, social status, or body size. In this godwit population, we have observed no sex differences in migration timing, but molt strategies vary dramatically both within and between sexes [22], [23]. This is reflected in the large span of ‘additional’ variation in timing of pre-breeding molt (Figure 4); some males initiate this molt more than two months prior to departure from New Zealand, whereas some females do not appear to initiate molt until after arrival on northbound staging grounds in the Yellow Sea [22]. Amid this range of strategies, individuals are relatively faithful to their own schedules, demonstrating how temporal constraints can manifest differently at the individual and population levels.

A striking finding is the almost complete absence of ‘additional’ variation during migration to the breeding grounds (Figure 4). It is generally observed that individuals vary in migratory performance (e.g., ‘low-quality’ individuals migrating later), but that sub-optimal performance will have a direct fitness cost, either in reproductive output or survival [4], [15], [54]. Our results suggest that such effects are negligible in this system; after accounting for breeding latitude, we observed essentially no inter-individual variation in how the journey to the breeding grounds was scheduled. This suggests: (1) low-quality or poor-condition individuals are rare in this population; (2) such individuals simply do not migrate; or (3) they prioritize migration timing, such that poor quality is instead reflected in, for example, body mass at departure or poor reproductive effort. During our study, no marked adult godwits ever failed to migrate in a particular year, and we were unable to link non-breeding performance with probability of returning the following year [23].

### Problems with Repeatability

With increasing interest in the flexibility of organisms in variable circumstances (e.g., climate change), there is a growing desire to describe consistency of individual behavior, and to make direct comparisons within and among studies. In bird migration studies, repeatability (intra-class correlation coefficient, *r* [16]) has become standard for describing the consistency with which individuals perform, but it is not precisely a measure of that; rather, it indicates how consistently individuals differ from each other. This is the variable of interest in some studies (e.g., as the upper bound of heritability [16]; but see [55]). However, it unfortunately tells us little about *absolute* consistency, which may be of greater interest to many phenology studies. The problem is that *r* combines population and individual variation to arrive at one value, and thus obscures two parameters that describe variation at different scales. Consequently, when a number of viable alternative strategies exist in a population, *r* is naturally inflated, regardless of the consistency of individual behavior. This renders *r* values essentially incomparable, even among parameters within a single study.

In our data, the problem is exemplified by the repeatability of pre-breeding molt initiation among all color-banded godwits (Table 1). The *r* value of 0.91 implies extraordinary consistency, but in fact individual variation in this parameter is, on average, greater than in migratory arrival and departure, which counter-intuitively show lower *r* values. This apparent paradox results from the relatively high population variation in pre-breeding molt initiation; due to a wide range of molt strategies in the population [22], individuals are more consistently different from each other while being less consistent individually than in other parameters. For this reason, we propose that authors should not report *r* values without presenting accompanying descriptions of absolute variation at the population and individual levels, as we have in Figure 2. This will be a step toward understanding the biological significance of within- and between-study differences in repeatability.

Our data also demonstrate the difficulty of comparing *r* values derived from different sample sizes. Both our geolocator-only and larger color-banded samples indicate that molts in New Zealand are generally less repeatable than movements, but three parameters with non-significant *r* values in one dataset appeared highly repeatable in the other (Table 1). Beyond the obviously greater statistical power afforded by larger samples when effect sizes are identical, *r* values also naturally increase as more of the total population variation is described in the sample (because *r* is driven by the ratio of population to individual variation). This is a particular problem for comparing *r* values currently available in migration literature, because multi-year individual studies using geolocation and satellite-telemetry typically contain small samples [20], [41] that cannot describe total population variation as completely as studies based on large samples of marked individuals [18], [56].

### Future Directions

The aim of most contemporary studies of temporal variation is to ascertain the capacity of organisms to address stochastic or directional environmental change [57]. Year-round comparative studies among species along a continuum of time and energy constraints would be a step toward understanding flexibility inherent in systems and identifying populations and specific annual stages prone to evolutionary constraints on adaptive phenological responses. For migratory birds, there has been a general lack of relevant long-term studies; due to the logistical difficulties of tracking individual migrants, few studies have contained individual data spanning more than 2–3 years. However, advances in satellite- and geolocator-tracking are making such studies feasible and, we propose, a priority. Because both individual behavior and environmental conditions are more likely to be consistent in consecutive years than over longer periods [58], we expect increases in both individual and population variation with additional years of data. However, the two may change disproportionately, depending on the scale at which time constraints operate. For example, a long-term increase in population but not individual variation could indicate that plastic responses to changing selection pressures are limited. In addition, the extent of increase in overall variation should vary among annual events, depending on control mechanisms; this may help identify life-history stages most prone to critical timing mismatches (e.g., [38]).

## References

[1] Alerstam T, Lindström Å (1990) Optimal bird migration: the relative importance of time, energy and safety. In: Gwinner E, editor. Bird Migration: Physiology and Ecophysiology. Berlin: Springer-Verlag. 331–351.

[2] Dawson A (2008) Control of the annual cycle in birds: endocrine constraints and plasticity in response to ecological variability. Philosophical Transactions of the Royal Society of London, Series B 363: 1621–1633. 18048294 10.1098/rstb.2007.0004 PMC2606722

[3] Jacobs JD, Wingfield JC (2000) Endocrine control of life-cycle stages: a constraint on response to the environment? Condor 102: 35–51.

[4] Møller AP (1994) Phenotype-dependent arrival time and its consequences in a migratory bird. Behavioral Ecology and Sociobiology 35: 115–122.

[5] Bety J, Giroux JF, Gauthier G (2004) Individual variation in timing of migration: causes and reproductive consequences in greater snow geese (*Anser caerulescens atlanticus*). Behavioral Ecology and Sociobiology 57: 1–8.

[6] Harrison XA, Blount JD, Inger R, Norris DR, Bearhop S (2011) Carry-over effects as drivers of fitness differences in animals. Journal of Animal Ecology 80: 4–18. 20726924 10.1111/j.1365-2656.2010.01740.x

[7] Studds CE, Marra PP (2007) Linking fluctuations in rainfall to nonbreeding season performance in a long-distance migratory bird, *Setophaga ruticilla* . Climate Research 35: 115–122.

[8] McNamara JM, Welham RK, Houston AI (1998) The timing of migration within the context of an annual routine. Journal of Avian Biology 29: 416–423.

[9] Farmer AH, Wiens JA (1999) Models and reality: time-energy trade-offs in pectoral sandpiper (*Calidris melanotos*) migration. Ecology 80: 2566–2580.

[10] Warnock N, Takekawa JY, Bishop MA (2004) Migration and stopover strategies of individual dunlin along the Pacific coast of North America. Canadian Journal of Zoology 82: 1687–1697.

[11] Madsen J (2001) Spring migration strategies in Pink-footed Geese *Anser brachyrhynchus* and consequences for spring fattening and fecundity. Ardea 89: 43–55.

[12] Nussey DH, Postma E, Gienapp P, Visser ME (2005) Selection on heritable phenotypic plasticity in a wild bird population. Science 310: 304–306. 16224020 10.1126/science.1117004

[13] Gwinner E (1996) Circannual clocks in avian reproduction and migration. Ibis 138: 47–63.

[14] Marra PP, Francis CM, Mulvihill RS, Moore FR (2005) The influence of climate on the timing and rate of spring bird migration. Oecologia 142: 307–315. 15480801 10.1007/s00442-004-1725-x

[15] Kokko H (1999) Competition for early arrival in migratory birds. Journal of Animal Ecology 68: 940–950.

[16] Nakagawa S, Schielzeth H (2010) Repeatability for Gaussian and non-Gaussian data: a practical guide for biologists. Biological Reviews 85: 935–956. 20569253 10.1111/j.1469-185X.2010.00141.x

[17] Pulido F (2007) Phenotypic changes in spring arrival: evolution, phenotypic plasticity, effects of weather and condition. Climate Research 35: 5–23.

[18] Battley PF (2006) Consistent annual schedules in a migratory shorebird. Biology Letters 2: 517–520. 17148277 10.1098/rsbl.2006.0535 PMC1833993

[19] Lourenço PM, Kentie R, Schroeder J, Groen NM, Hooijmeijer JCEW, et al (2011) Repeatable timing of northward departure, arrival and breeding in Black-tailed Godwits *Limosa l. limosa*, but no domino effects. Journal of Ornithology 152: 1023–1032.

[20] Vardanis Y, Klaassen RHG, Strandberg R, Alerstam T (2011) Individuality in bird migration: routes and timing. Biology Letters 7: 502–505. 21307045 10.1098/rsbl.2010.1180 PMC3130220

[21] Conklin JR, Battley PF, Potter MA, Fox JW (2010) Breeding latitude drives individual schedules in a trans-hemispheric migrant bird. Nature Communications 1: article 67 doi: 10.1038/ncomms1072 10.1038/ncomms1072 20842198

[22] Conklin JR, Battley PF (2011) Contour feather moult of bar-tailed godwits (*Limosa lapponica baueri*) in New Zealand and the Northern Hemisphere reveals multiple strategies by sex and breeding region. Emu 111: 330–340.

[23] Conklin JR, Battley PF (2012) Carry-over effects and compensation: late arrival on non-breeding grounds affects wing moult but not plumage or schedules of departing bar-tailed godwits. Journal of Avian Biology 43: 252–263.

[24] Gill RE Jr, Tibbitts TL, Douglas DC, Handel CM, Mulcahy DM, et al (2009) Extreme endurance flights by landbirds crossing the Pacific Ocean: ecological corridor rather than barrier? Proceedings of the Royal Society of London, Series B 276: 447–457. 18974033 10.1098/rspb.2008.1142 PMC2664343

[25] Battley PF, Warnock N, Tibbitts TL, Gill RE Jr, Piersma T, et al (2012) Contrasting extreme long-distance migration patterns in bar-tailed godwits *Limosa lapponica* . Journal of Avian Biology 43: 21–32.

[26] Conklin JR, Battley PF (2011) Impacts of wind on individual migration schedules of New Zealand bar-tailed godwits. Behavioral Ecology 22: 854–861.

[27] Conklin JR (2011) Extreme migration and the annual cycle: individual strategies in New Zealand Bar-tailed Godwits. PhD thesis, Massey University, New Zealand. 209 pp.

[28] Fox JW (2010) Geolocator Manual, Version 8. British Antarctic Survey.

[29] Eichhorn G, Afanasyev V, Drent RH, van der Jeugd HP (2006) Spring stopover routines in Russian barnacle geese *Branta leucopsis* tracked by resightings and geolocation. Ardea 94: 667–678.

[30] Lessells CM, Boag PT (1987) Unrepeatable repeatabilities: a common mistake. Auk 104: 116–121.

[31] Becker WA (1984) A Manual of Quantitative Genetics. Pullman, Washington: Academic Enterprises.

[32] National Oceanic and Atmospheric Administration (NOAA) National Climatic Data Center. Available: http://www.ncdc.noaa.gov/snow-and-ice/snow-cover.php. Accessed 2012 Jun.

[33] McCaffery BJ, Gill RE Jr (2001) Bar-tailed godwit (*Limosa lapponica*). In: Poole A, Gill F, editors. The Birds of North America. Philadelphia: The Birds of North America, Inc.

[34] Bell AM, Hankison SJ, Laskowski KL (2009) The repeatability of behaviour: a meta-analysis. Animal Behaviour 77: 771–783. 24707058 10.1016/j.anbehav.2008.12.022 PMC3972767

[35] Lyons JE, Haig SM (1995) Fat content and stopover ecology of spring migrant semipalmated sandpipers in South Carolina. Condor 97: 427–437.

[36] Warnock N, Bishop MA (1998) Spring stopover ecology of migrant western sandpipers. Condor 100: 456–467.

[37] Battley PF, Piersma T, Rogers DI, Dekinga A, Spaans B, et al (2004) Do body condition and plumage during fuelling predict northwards departure dates of great knots *Calidris tenuirostris* from north-west Australia? Ibis 146: 46–60.

[38] Both C, Visser ME (2001) Adjustment to climate change is constrained by arrival date in a long-distance migrant bird. Nature 411: 296–298. 11357129 10.1038/35077063

[39] Smith PA, Gilchrist HG, Forbes MR, Martin J, Allard K (2010) Inter-annual variation in the breeding chronology of arctic shorebirds: effects of weather, snow melt and predators. Journal of Avian Biology 41: 292–304.

[40] Fransson T (1995) Timing and speed of migration in North and West European populations of *Sylvia* warblers. Journal of Avian Biology 26: 39–48.

[41] Alerstam T, Hake M, Kjellén N (2006) Temporal and spatial patterns of repeated migratory journeys by ospreys. Animal Behaviour 71: 555–566.

[42] Egevang C, Stenhouse IJ, Phillips RA, Petersen A, Fox JW, et al (2010) Tracking of Arctic terns *Sterna paradisaea* reveals longest animal migration. Proceedings of the National Academy of Sciences 107: 2078–2081. 10.1073/pnas.0909493107 PMC2836663 20080662

[43] Studds CE, Marra PP (2011) Rainfall-induced changes in food availability modify the spring departure programme of a migratory bird. Proceedings of the Royal Society of London, Series B 278: 3437–3443. 21450737 10.1098/rspb.2011.0332 PMC3177634

[44] Barshep Y, Minton C, Underhill LG, Remisiewicz M (2011) The primary moult of Curlew Sandpipers *Calidris ferruginea* in north-western Australia shifts according to breeding success. Ardea 99: 43–51.

[45] Weber TP, Ens BJ, Houston AI (1998) Optimal avian migration: a dynamic model of fuel stores and site use. Evolutionary Ecology 12: 377–401.

[46] Holmgren N, Hedenström A (1995) The scheduling of molt in migratory birds. Evolutionary Ecology 9: 354–368.

[47] Liechti F (2006) Birds: blowin’ by the wind? Journal of Ornithology 147: 202–211.

[48] Dawson A, Hinsley SA, Ferns PN, Bonser RHC, Eccleston L (2000) Rate of moult affects feather quality: a mechanism linking current reproductive effort to future survival. Proceedings of the Royal Society of London, Series B 267: 2093–2098. 11416914 10.1098/rspb.2000.1254 PMC1690790

[49] Helm B, Gwinner E (2006) Timing of molt as a buffer in the avian annual cycle. Acta Zoologica Sinica 52 (suppl.): 703–706.

[50] Noskov G, Rymkevich T, Iovchenko N (1999) Intraspecific variation of moult: adaptive significance and ways of realisation. In: Adams N, Slotow R, editors. Proceedings of the 22nd International Ornithological Congress, Durban. Johannesburg: BirdLife South Africa. 544–563.

[51] Dawson A (2006) Control of molt in birds: association with prolactin and gonadal regression in starlings. General and Comparative Endocrinology 147: 314–322. 16530194 10.1016/j.ygcen.2006.02.001

[52] Hahn TP, Swingle J, Wingfield JC, Ramenofsky M (1992) Adjustments of the prebasic molt schedule in birds. Ornis Scandinavica 23: 314–321.

[53] Mitchell GW, Wheelwright NT, Guglielmo CG, Norris DR (2012) Short- and long-term costs of reproduction in a migratory songbird. Ibis 154: 325–337.

[54] Drent R, Both C, Green M, Madsen J, Piersma T (2003) Pay-offs and penalties of competing migratory schedules. Oikos 103: 274–292.

[55] Dohm MR (2002) Repeatability estimates do not always set an upper limit to heritability. Functional Ecology 16: 273–280.

[56] Rees EC (1989) Consistency in the timing of migration for individual Bewick’s swans. Animal Behaviour 38: 384–393.

[57] Walther G-R, Post E, Convey P, Menzel A, Parmesan C, et al (2002) Ecological responses to recent climate change. Nature 416: 389–395. 11919621 10.1038/416389a

[58] Catry P, Ruxton GD, Ratcliffe N, Hamer KC, Furness RW (1999) Short-lived repeatabilities in long-lived great skuas: implications for the study of individual quality. Oikos 84: 473–479.

